# Relationships between Perceived Discrimination and Suicidal Ideation among Impoverished Chinese College Students: The Mediating Roles of Social Support and Loneliness

**DOI:** 10.3390/ijerph19127290

**Published:** 2022-06-14

**Authors:** Yanxia Mao, Luming Liu, Zi’ang Lu, Wenchao Wang

**Affiliations:** 1 School of Education Science, Luoyang Normal University, Luoyang 471934, China; maoyanxia@lynu.edu.cn; 2 Beijing Key Laboratory of Applied Experimental Psychology, National Demonstration Center for Experimental Psychology Education (Beijing Normal University), Faculty of Psychology, Beijing Normal University, Beijing 100875, China; 201911061159@mail.bnu.edu.cn (L.L.); 202011061067@mail.bnu.edu.cn (Z.L.)

**Keywords:** impoverished college students, perceived discrimination, social support, loneliness, suicidal ideation

## Abstract

We explored the mediating effect of social support and loneliness in the relationships between perceived discrimination and suicidal ideation among impoverished Chinese college students. Using the convenience cluster sampling method, we chose a total of 964 impoverished college students from a central province of China. Students completed the cross-sectional survey using the Perceived Discrimination Questionnaire, the Social Support Rating Scale, the University of California at Los Angeles Loneliness Scale, and the Beck Scale for Suicidal Ideation. Correlation analysis and structural equation modeling analysis were conducted to clarify the relationships between study variables. Correlation analysis showed that perceived discrimination, loneliness, and suicidal ideation were positively correlated with each other; social support was negatively correlated with perceived discrimination, loneliness, and suicidal ideation. In addition, structural equation modeling analysis indicated that perceived discrimination had a direct positive effect on suicidal ideation; social support and loneliness partially mediated the relationship between perceived discrimination and suicidal ideation. Specifically, perceived discrimination was positively associated with suicidal ideation via social support and loneliness separately, and had a serial association through both social support and loneliness. Thus, perceived discrimination may have influenced suicidal ideation through both social support and loneliness.

## 1. Introduction

In recent years, the suicide rate among college students ((1.55~2.56)/100,000) maintained an upward trend in general and was higher than non-college students at the same age ((0.49~1.43)/100,000) [1,2]. Meanwhile, as the largest developing country globally, China has a large number of impoverished people, and the mental health of the impoverished population in China needs attention. Impoverished college students have both the identity of college students and impoverished students. Due to social comparison, impoverished students tend to have adverse psychological feelings and behavioral changes. Thus, the suicide-related behaviors of Chinese impoverishment are worth noticing.

### 1.1. Basic Situation of Chinese Impoverished College Students and Their Suicidal Ideation

In China, economically disadvantaged students generally refer to college students enrolled in the state plan who are unable to pay the basic expenses of their studies and live on funds raised by themselves and their families [3]. They can apply for national grant-aided loans to temporarily cover the tuition or apply for a tuition waiver. The nation and universities also provide subsidies and scholarships to impoverished students each term. In addition, universities provide part-time positions on campus. Therefore, in most situations, impoverished students’ requirements for tuition can be satisfied. However, the income above may not ensure the same life quality as general students for daily life expenses. Since campus life can be regarded as a miniature society, the different consumption ideas caused by income differences may lead to discrimination and conflicts.

According to the strain theory of suicide, the deprivation strain from poverty can be a direct source of suicide [4]. Empirical studies also show that rural college students with low socioeconomic status are more likely to have suicidal ideation [5]. According to a cross-national epidemiological investigation, suicidal ideation is an important predictor of suicidal behavior, and one-third of the population with suicidal ideation will eventually develop suicidal behavior [5,6]. A similar result was also found in the Chinese adolescent group [7]. The current study intends to explore the psychological factors affecting the occurrence of suicidal ideation among impoverished college students.

### 1.2. Perceived Discrimination Affects Suicidal Ideation

In China, impoverished students tend to perceive a higher extent of discrimination [8,9]. At the individual level, due to the gap in their economic conditions, they may fail to reach the same life quality as other students, which may increase their perceived discrimination when interacting with others. Society tends to prejudice and stigmatize impoverished people at the group level due to cultural factors [10]. For example, some impoverished people pursue immediate interests of their own by improper means or foul, which is seen as selfish and immoral in Chinese collectivist culture. Since the word poverty is often associated with negative images, impoverished students usually try to avoid or hide this identity and even refuse to receive subsidies [8]. These subjective experiences generated by different attitudes between the group are referred to as perceived discrimination [11]. According to the integrated motivational–volitional model proposed by O’Connor, a sense of humiliation is a leading factor in suicidal ideation [12], and discrimination plays a critical role between poverty and psychological health [13]. Of note, the Schematic Appraisals Model of Suicide (SAMS) suggests that perceived discrimination is a belief that embodies a cognitive process of self-appraisal and directly leads people to have a more negative, hostile perception of the outside world [14]. In other words, a perception of discrimination represents an individual’s negative psychological feeling of humiliation, regardless of whether others have truly discriminated against them [15]. Several empirical studies have found that perceived discrimination leads to an increased risk of suicidal ideation [16,17,18].

### 1.3. The Mediating Role of Social Support and Loneliness

Although one’s perception of discrimination is considered an important risk factor for suicidal ideation, the internal mechanisms are not well understood. According to the interpersonal theory of suicide, suicidal ideation arises from two interpersonal states—perceived burdensomeness and thwarted belongingness. Perceived burdensomeness is an externally oriented factor, referring to an individual’s false perception that their inability will burden their friends, family, or society. Thwarted belongingness is an internally oriented factor, referring to the state of psychological distress caused when the basic need to connect with others is not met [19]. When interpersonal needs are not met, individuals may feel lonely due to the lack of social connection. These negative psychological feelings will further intensify the risk of suicidal ideation [20].

For the externally oriented factor, according to the dynamic effect model of social support, stressors affect the acquisition and use of social support, and some special stressors play a vital role in predicting social support [21]. Perceiving that others’ discrimination against oneself will cause huge psychological pressure may make it difficult for one to seek and make full use of social support actively. Empirical studies have found that discrimination can directly affect people’s social support and, subsequently, indirectly affect their physical and mental health [22]. Meanwhile, a higher level of social support may alleviate perceived burdensomeness [23], which could reduce the level of suicidal ideation [20].

Meanwhile, loneliness is an internal emotional factor that impacts individual suicidal ideation. Loneliness refers to the experience of feeling lonely, helpless, and depressed when one’s social needs are not met [24], which is an important indicator in predicting suicidal ideation [25,26]. Loneliness may also act as the mediator between perceived discrimination and suicidal ideation [27]. Further, according to the rational emotive therapy proposed by Ellis, people’s loneliness is largely caused by unreasonable cognition [28]. Impoverished students who perceive that they are discriminated against will feel excluded and treated differently by others, thus resulting in loneliness. Hence, social support may also predict loneliness.

Based on the discussion above, social support and loneliness might mediate the effects of perceived discrimination on suicidal ideation; a chain mediation structure among perceived discrimination, social support, loneliness, and suicidal ideation may also exist.

### 1.4. The Present Study

In summary, although some previous studies have investigated the effect of perceived discrimination on the mental and physical health of impoverished students and its mechanisms, few studies have examined how perceived discrimination affects their suicidal ideation. Students with financial difficulties account for approximately 20% to 30% of all college students in China [29] and comprise a large special group in colleges that cannot be ignored. Based on previous studies and relevant theories, we proposed the following hypotheses: perceived discrimination positively predicts suicidal ideation in impoverished students, and social support and loneliness play mediating roles in the influence of perceived discrimination on suicidal ideation. Perceived discrimination manifests its role in suicidal ideation through the serial mediation of social support and loneliness.

## 2. Methods

### 2.1. Participants

This study was surveyed in May 2018 at a regular university in Hubei Province with more than 20,000 undergraduate students from all provinces in China. With the permission of the university’s president, the student administration of the university assisted us in contacting the class teachers. Impoverished students from 20 classes of each grade were randomly selected to conduct the survey. The survey was conducted online, and the participants filled in the questionnaire through Wenjuanxing. The inclusion criteria were as follows: (1) being recognized as having economic difficulties by their village or community committee; (2) receiving national grant-aided loans awarded by the university; and (3) having no recent serious traumatic events and no mental illness or poor health history. Thirty-six invalid samples were deleted based on the answering duration (e.g., less than 5 min) and answer quality (e.g., giving all items the same answer). After the screening process, we received a total of 964 valid questionnaires, with 438 males (45.4%) and 526 females (54.6%), ranging in age from 18 to 24 (*M* = 20.28 years, *SD* = 1.79), see Table 1 for more information about the participants. We explained the survey’s purpose and obtained informed consent from the participating students. The study was approved by the research ethics committee of the first author’s institution, which confirmed that all research processes performed followed ethical standards.

### 2.2. Materials

#### 2.2.1. Perceived Discrimination

We used the sub-questionnaire for individually perceived discrimination—the Perceived Discrimination Questionnaire—to assess the level of participants’ individually perceived discrimination. The questionnaire showed good validity and reliability among Chinese college students [8]. The sub-questionnaire for individually perceived discrimination has three questions and is rated on a 5-point Likert-type scale ranging from strongly disagree (1) to strongly agree (5). In the current study, Cronbach’s α was 0.83.

#### 2.2.2. Social Support

We used the Chinese version of the Social Support Rating Scale (SSRS) to assess the level of participants’ social support over the past year. The scale is divided into three dimensions: subjective support, objective support, and the utilization of support. It showed good validity and reliability among the Chinese population [30]. This scale consists of 10 items, and higher scores reflect a higher level of social support. In the current study, Cronbach’s α was 0.89.

#### 2.2.3. Loneliness

We assessed loneliness using the Chinese version of the University of California at Los Angeles Loneliness Scale, which showed good validity and reliability among the Chinese population [31]. The scale has 20 items and is rated on a 4-point Likert-type scale ranging from strongly disagree (1) to strongly agree (4). Higher scores indicate higher levels of loneliness. The Cronbach’s α was 0.87. The total score of the scale greater than 44 indicates strong loneliness, the total score less than 28 indicates a weak sense of loneliness, and the total score between 29 and 43 indicates a moderate level of loneliness.

#### 2.2.4. Suicidal Ideation

We assessed suicidal ideation using the Chinese version of the Beck Scale for Suicidal Ideation (BSSI) [32], which showed good validity and reliability among the Chinese population [31]. The scale is a 19-item self-rated questionnaire consisting of two subscales, suicidal ideation, and suicidal tendency, with questions 1–5 to assess suicidal ideation and questions 6–19 to assess suicidal tendency. The current study used questions 1–5 to assess suicidal ideation. Items are rated on a 3-point Likert-type scale ranging from strongly disagree (0) to strongly agree (2), with higher scores reflecting more intense thoughts about suicide. Participants were considered to have suicidal ideation if they scored anything other than 0 on questions 4 or 5 [32]. In the present study, the Cronbach’s α coefficient for the scale was 0.82.

### 2.3. Data Analysis

We conducted statistical analyses using IBM SPSS 16.0 and AMOS 17.0 software. We handled missing data with full information maximum likelihood estimates (FIMLs) in structural equation models (SEMs). The results of Harman’s single-factor test suggest that the variance for both rotated and unrotated first factors was below the threshold of 40%, which means there was no significant common method bias in the study samples. The Bollen-Stine Bootstrapping method is adopted to test the mediation effect, which is regarded as an effective method to deal with non-normal data [33]. Since social support and suicidal ideation contains multiple dimensions, we constructed them as latent variables by their subdimensions. Firstly, the fitness of the measurement model was tested. Then, the direct effect model between perceived discrimination and suicidal ideation and the indirect model were analyzed. We used the following four indices to evaluate the model’s goodness of fit: chi-square values, the comparative fit index (CFI), the Tucker–Lewis index (TLI), and the root mean square error of approximation (RMSEA). The general cut-offs for accepting a model are equal to or greater than 0.90 for the CFI and TLI, and less than 0.08 for the RMSEA.

## 3. Results

### 3.1. Descriptive Statistics and Correlations

Table 1 shows the participants’ characteristics, and of the 964 college students surveyed, 195 (20.22%) reported suicidal ideation in the past week. As can be seen from the total score of loneliness, 379 (39.32%) college students had high loneliness level, 472 (48.96%) had moderate loneliness level, and 113 (11.73%) had low loneliness level. In the structural equation model analyses, gender, grade, household registration, and poverty level were taken as covariates.

Table 2 presents the descriptive statistics of the study variables and the bivariable correlations between them. According to the results of the Pearson correlation, there was a significant negative correlation between perceived discrimination and social support (r = −0.31, *p* < 0.001), and a significant positive correlation between perceived discrimination and loneliness and suicidal ideation (r = 0.37, *p* < 0.001; r = 0.26, *p* < 0.001). Social support was negatively correlated with loneliness and suicidal ideation (r = −0.36, *p* < 0.001; r = −0.22, *p* < 0.001), and loneliness was positively correlated with suicidal ideation (r = 0.32, *p* < 0.001).

### 3.2. Structural Equation Model Analyses

**Figure 1:**
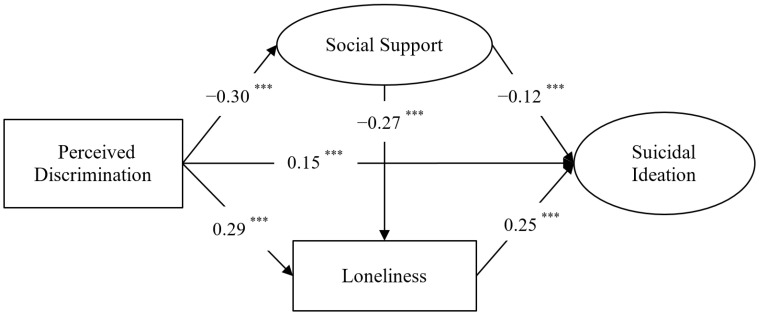
Multiple indirect effects model. Note: All path coefficients were standardized coefficients. *** *p* < 0.001.

In the first stage, we established a measurement model consisting of eight observational variables and two potential factors (social support and suicidal ideation), and the fit was acceptable: *χ*^2^/*df* = 4.15, CFI = 0.945, TLI = 0.922, RMSEA (90% CI) = 0.062 (0.046–0.078). The load of each factor was significant (*p* < 0.001) to the potential variables index. In the second stage of analysis, after controlling for gender, grade, household registration, and poverty level, we built a direct effect model that demonstrated the direct relation of perceived discrimination on suicidal ideation; the results showed that the model fit well: *χ*^2^/*df* = 3.96, CFI = 0.928, TLI = 0.934, RMSEA (90% CI) = 0.051 (0.039~0.064). Perceived discrimination positively associated with suicidal ideation (*β* = 0.28 (0.23~0.34), *p* < 0.001). On the basis of the direct effect model, we included social support and loneliness to establish a mediation model, which also fit well: *χ*^2^/*df* = 3.23, CFI = 0.962, TLI = 0.935, RMSEA (90% CI) = 0.045 (0.027~0.062). The model showed that perceived discrimination negatively related to social support (*β* = −0.30, *p* < 0.001) and positively related to loneliness and suicidal ideation (*β* = 0.29, *p* < 0.001; *β* = 0.15, *p* < 0.001). Social support negatively associated with loneliness and suicidal ideation (*β* = −0.27, *p* < 0.001; *β* = −0.12, *p* < 0.001), and loneliness positively associated with suicidal ideation (*β* = 0.25, *p* < 0.001). See Figure 1.

Next, we used the bias-corrected bootstrap procedure (5000 random samples) to test the mediating effect. Table 3 portrays the results. The 95% CIs of the mediating effect of each pathway did not contain 0, indicating the existence of the mediating effect.

## 4. Discussion

### 4.1. The Direct Effect of Perceived Discrimination on Suicidal Ideation

We built a SEM to investigate the influencing mechanism of perceived discrimination on suicidal ideation among impoverished students. The results show that perceived discrimination directly positively predicted suicidal ideation. According to the integrated motivational–volitional model of suicidal behavior, the reason why an individual has suicidal ideation is largely due to their sense of humiliation [11]. Due to their financial constraints, impoverished students are relatively deficient in material resources. In the collective life of the university, there are often material comparisons among students, and impoverished students will inevitably feel inferior. In addition, when applying for educational grants, some universities require impoverished students to expose their families’ financial difficulties to their classmates. This process will place great psychological pressure on impoverished students, leading to their perception of discrimination. Notably, the perceived discrimination in this study was subjective, which might be mixed with unreasonable cognition [34]. However, regardless of whether discrimination objectively exists, as long as impoverished students have a high level of perceived discrimination, their suicidal ideation will intensify accordingly.

### 4.2. The Mediating Effect of Social Support and Loneliness

We also found that perceived discrimination may influence the suicidal ideation of impoverished students through externally oriented factors and internally oriented factors (i.e., social support and loneliness), which verified the interpersonal theory of suicide.

According to the dynamic effect model of social support, interpersonal communication is a basic psychological requirement for individuals, and others’ support and company could alleviate the suicidal ideation raised by unpleasant events [20]. In addition, the perception of social support is affected by special stressors, and social support can be an important environmental factor influencing individuals’ suicidal ideation [21]. That is, for impoverished students, perceiving others’ discrimination makes them feel reluctant to engage in social activities, which may cause a low level of perceived social support and eventually increase their suicidal ideation.

In addition to social support, we found that loneliness can mediate the relationship between perceived discrimination and suicidal ideation. According to the definition, perceived discrimination is a feeling caused by being ostracized and treated differently by the mainstream. Others’ real discrimination or the improper, subjective, perceived discrimination of impoverished students might cause them to become alienated from their classmates, leading to exacerbated loneliness [24]. Moreover, high loneliness will reduce the individual’s ability to cope with stressful events actively, produce a series of adverse psychological reactions, and lead to suicidal ideation [35].

Further, we found that the perception of discrimination could increase suicidal ideation through a serial mediation of social support and loneliness. On one hand, social support is an important psychosocial resource that can protect individuals’ mental and physical health in stressful situations, such as reducing loneliness [29]. On the other hand, individuals with high social support generally have better interpersonal communication status, and the satisfaction of interpersonal communication needs will further reduce individual loneliness and suicidal ideation [36].

### 4.3. Implications and Limitations

Overall, we examined the mechanisms of impoverished college students’ perceived discrimination on suicidal ideation. The results verified the integrated motivational–volitional model and the interpersonal theory of suicide; this suggests that we should pay attention to the feeling of being discriminated against among impoverished college students and their interpersonal needs. All sectors of society should help impoverished students to establish positive mental attitudes to mitigate perceived discrimination, improve social support, and eliminate loneliness, eventually reducing suicidal ideation. Specifically, aid institutions of colleges are supposed to provide impoverished students with not only financial support but also psychological support such as mental health courses [37]. For example, universities should aim at breaking the stereotype and focusing on the positive qualities of impoverished students to help them gain more social support. In addition, they should guide impoverished students to participate in societies, associations, and work–study positions to make them perceive less loneliness. Psychological assistance is also needed to help some of impoverished students to establish confidence instead of negative psychological states like self-abasement [38].

However, there are also some limitations in this study. First, we did not further subdivide the sources of social support, and different sources of social support may have different effects on suicidal ideation. Second, although suicidal ideation is an important predictor of suicidal behavior, whether an individual with suicidal ideation will die by suicide is also influenced by impulsivity, pain tolerance, and other factors [39]. Future research can explore the mediating effect of other factors on perceived discrimination’s impact on suicidal ideation among impoverished students. Third, the perceived discrimination level was only investigated in impoverished students, which meant the lack of a control group. The discrimination from other causes still could not be entirely excluded. Follow-up studies can further investigate the discrimination from other sources and their consequence on individuals to highlight the effects of discrimination resulting from poverty. Furthermore, the cross-sectional nature prevented us from establishing verifiable causality, and the convenience sampling method could not ensure the unbiasedness of the participant group. In future studies, more in-depth investigation of the psychological mechanisms affecting the suicidal behavior of impoverished students can occur.

## 5. Conclusions

The present study verified that perceived discrimination could be a significant risk factor for impoverished Chinese college students’ suicidal ideation. Specifically, social support and loneliness were two key underlying mechanisms. That is, perceived discrimination could reduce one’s social support and increase one’s loneliness, which eventually results in suicidal ideation. Thus, clinicians and social workers at college may focus on external supports and internal feelings/cognitions simultaneously for suicide prevention.

## Figures and Tables

**Table 1:** Participants’ characteristics.

	*n*	%
**Gender**		
Male	438	45.4
Female	526	54.6
**Grade**		
Grade one	248	25.7
Grade two	255	26.5
Grade three	273	28.3
Grade four	188	19.5
**Household registration**		
Urban	169	17.5
Rural	795	82.5
**Poverty level** *		
Extremely	127	13.2
Ordinary	837	86.8

Note: * According to the criteria for identifying impoverished students, extremely poor students need to meet at least one of the following criteria. (1) Living in a state-level poverty-stricken county, both parents are chronically ill and need long-term self-funded treatment or have a severe disability, resulting in weak working ability or loss of working ability. (2) Orphans and disabled students, children of martyrs, and other families in extreme economic difficulties. (3) Both parents are laid off, and both parents have been seriously ill for a long time and need long-term self-pay treatment or have a severe disability, resulting in weak labor ability or loss of labor ability. Ordinary impoverished students need to meet at least one of the following criteria. (1) Living in a state-level poverty-stricken county, one of the parents is chronically ill and needs long-term self-funded treatment or has a severe disability, resulting in weak working ability or loss of working ability. (2) Both parents are laid off, and one of the parents has been seriously ill for a long time and needs long-term self-pay treatment or has a severe disability, resulting in weak labor ability or loss of labor ability. (3) Children from single-parent families without financial resources.

**Table 2:** Bivariate correlations and descriptive statistics among study variables.

	*M ± SD*	1	2	3	4
1. Perceived Discrimination	8.58 ± 2.78	-			
2. Social Support	43.53 ± 16.34	−0.31 ***	-		
3. Loneliness	51.38 ± 8.43	0.37 ***	−0.36 ***	-	
4. Suicidal ideation	0.95 ± 0.82	0.26 ***	−0.22 ***	0.32 ***	-

Note: *** *p* < 0.001.

**Table 3:** Bias-corrected bootstrap text on mediating effects.

Indirect Paths	Standardized *β*	Standardized 95% CI	
		Low	High
PD–SS–SI	0.046 *	0.025	0.077
PD–Loneliness–SI	0.075 **	0.047	0.103
PD–SS–Loneliness–SI	0.016 *	0.011	0.021

Note: All path coefficients were standardized coefficients. PD = perceived discrimination, SS = social support, SI = suicidal ideation. * *p* < 0.05, ** *p* < 0.01.

## Data Availability

The data that support the findings of this study are available from the corresponding author, up-on reasonable request.
